# Sustained inflations versus UK standard inflations during initial resuscitation of prematurely born infants in the delivery room: a study protocol for a randomised controlled trial

**DOI:** 10.1186/s13063-017-2311-y

**Published:** 2017-11-28

**Authors:** Katie A. Hunt, Kamal Ali, Theodore Dassios, Anthony D. Milner, Anne Greenough

**Affiliations:** 1 Division of Asthma, Allergy and Lung Biology, MRC & Asthma UK Centre in Allergic Mechanisms of Asthma, Kings College London, London, UK; 2 Neonatal Intensive Care Centre, King’s College Hospital NHS Foundation Trust, 4th Floor Golden Jubilee Wing, Denmark Hill, London, SE5 9RS UK; 3 NIHR Biomedical Research Centre at Guy’s and St Thomas’ NHS Foundation Trust and King’s College London, London, UK

**Keywords:** Resuscitation, Sustained inflation, Prematurely born infants

## Abstract

**Background:**

Many infants born at less than 34 weeks of gestational age will require resuscitation in the delivery suite. Yet, different resuscitation techniques are specified in different national guidelines, likely reflecting a limited evidence base. One difference is the length of mechanical inflation initially delivered to infants either via a facemask or endotracheal tube. Some guidelines specify short inflations delivered at rates of 40–60/min, others recommend initial inflations lasting 2–3 s or sustained inflations lasting for ≥ 5 s for initial resuscitation. Research has shown that tidal volumes > 2.2 mL/kg (the anatomical dead space) are seldom generated unless the infant’s respiratory effort coincides with an inflation (active inflation). When inflations lasting 1–3 s were used, the time to the first active inflation was inversely proportional to the inflation time. This trial investigates whether a sustained inflation or repeated shorter inflations is more effective in stimulating the first active inflation.

**Methods:**

This non-blinded, randomised controlled trial performed at a single tertiary neonatal unit is recruiting 40 infants born at < 34 weeks of gestational age. A 15-s sustained inflation is being compared to five repeated inflations of 2–3 s during the resuscitation at delivery. A respiratory function monitor is used to record airway pressure, flow, expiratory tidal volume and end tidal carbon dioxide (ETCO_2_) levels. The study is performed as emergency research without prior consent and was approved by the NHS London-Riverside Research Ethics Committee. The primary outcome is the minute volume in the first minute of resuscitation with secondary outcomes of the time to the first active inflation and ETCO_2_ level during the first minute of recorded resuscitation.

**Discussion:**

This is the first study to compare a sustained inflation to the current UK practice of five initial inflations of 2–3 s.

**Trial registration:**

ClinicalTrials.gov, NCT02967562. Registered on 15 November 2016.

**Electronic supplementary material:**

The online version of this article (doi:10.1186/s13063-017-2311-y) contains supplementary material, which is available to authorized users.

## Background

Seventy-five percent of infants born at < 34 weeks of gestational age require resuscitation including positive pressure ventilation and/or endotracheal intubation at delivery [1]. Current UK guidelines recommend initial resuscitation using five inflations of 2–3 s duration with peak inflation pressures of 20–25 cmH_2_O [2]. It has been shown, however, that despite resuscitation training, clinicians in both simulated and real resuscitation scenarios do not deliver the recommended duration of the inflations [3, 4]. This, combined with leaks around the facemask often as large as 50% or greater, contributes to low expired tidal volumes during resuscitation [5]. Indeed, during resuscitation by facemask or endotracheal tube, tidal volumes and end tidal carbon dioxide (ETCO_2_) levels remained low until a respiratory effort was made coinciding with a mechanical inflation (an active inflation) [3, 6]. Analysis of data collected during the resuscitation of infants born at < 34 weeks of gestational age who received inflations of 1–3 s in duration demonstrated that active inflations occurred sooner with longer inflation times [7].

There is evidence that sustained inflations (≥5 s) may be of benefit. In preterm rabbits, a sustained inflation of 20 s compared to a shorter inflation or no inflation before mechanical ventilation was commenced was associated with a more rapid establishment of a functional residual capacity; 90% of lung aeration was achieved within the first 14 s [8, 9]. Preterm lambs who received a 60-s sustained inflation followed by mechanical ventilation compared to those who received ventilation alone (tidal volumes of 7 mL/kg delivered at a rate of 60 bpm) had increased pulmonary blood flow, more stable cerebral oxygen delivery and better compliance during subsequent ventilation [10]. Comparison of five repeated 3-s inflations to a 20-s sustained inflation in asphyxiated lambs delivered near term showed that the sustained inflation provoked an earlier rise in heart rate, improved gas exchange and improved lung compliance during subsequent ventilation [11]. Another study in lambs, however, showed that a 30-s sustained inflation compared to no sustained inflation led to more cerebral vascular leakage and potential perturbation of blood–brain barrier function [12]. In all of the above studies, the lambs were sedated during resuscitation, whereas infants are not and indeed their respiratory efforts can make an important contribution to gas exchange [3, 6].

There have been several studies of sustained inflations in infants. Early studies in term infants born after elective Caesarean section demonstrated that a 5-s inflation produced a larger inflation volume than a 1-s inflation and also was more efficient at establishing a functional residual capacity [13, 14]. A study of infants born at < 32 weeks of gestational age demonstrated that those who were resuscitated with a sustained inflation compared to a historical cohort resuscitated without a sustained inflation were less likely to be intubated (51% vs 76%, *p* < 0.0001) and had lower durations of mechanical ventilation (5 vs 11 days, *p* < 0.008) and supplementary oxygen therapy (21 vs 31 days, *p* = 0.016) [15]. They were also less likely to receive postnatal steroids (25% vs 10%, *p* = 0.01) and had a lower incidence of bronchopulmonary dysplasia (BPD) (7% vs 25%, *p* = 0.04) [15]. Another study compared infants born at < 34 weeks of gestational age who received a 15-s sustained inflation to a historical cohort who received recurrent inflations at 60 breaths per minute. The sustained inflation group had a lower need for intubation (6% vs 21%, *p* < 0.01) and a shorter duration of ventilation (9.1 vs 13.8 days, *p* < 0.001) [16]. The findings of both studies [15, 16], however, should be interpreted with caution as other changes in perinatal care may have influenced the outcomes. In infants born at 25–33 weeks, a 10-s sustained inflation delivered via a nasopharyngeal tube compared to ventilation with initial inflation pressures of 30–40 cmH_2_O, then 20 cmH_2_O, at a rate of 60 bpm via a bag-mask device, was associated with a lower need for endotracheal intubation and mechanical ventilation, a shorter duration of total respiratory support and a lower rate of BPD. There were, however, limitations to the study, as the ‘ventilation’ group did not receive positive end expiratory pressure (PEEP) during resuscitation and were intubated and ventilated after 30 s of mask ventilation if unstable, whereas the sustained inflation group subsequently received nasal continuous positive airways pressure (CPAP) or several minutes of nasal ventilation as part of stabilization [17]. A randomised trial comparing a 15-s sustained inflation to resuscitation with inflations at 60 breaths per minute both delivered by nasopharyngeal tube, did not demonstrate significant differences in the need for mechanical ventilation. The trial, however, was stopped early due to slow recruitment [18]. In a subsequent randomised trial enrolling infants born at 25–29 weeks of gestational age, a 15-s sustained inflation followed by nasal CPAP was compared to nasal CPAP alone. A smaller proportion of the sustained inflation group required mechanical ventilation in the first 72 h after birth (53% vs 65%, *p* = 0.04) [19]. A further randomised trial enrolled infants born at 25–32 weeks of gestational age and randomised them to either a 15-s inflation at 25 cmH_2_O or resuscitation as per the American Heart Association guidelines (positive pressure ventilation at 15–20/5 cmH_2_O). A lower inspired oxygen concentration (FiO_2_) 10 min after delivery was demonstrated in the sustained inflation group (0.28 vs 0.47) (*p* < 0.01). Among those born < 28 weeks of gestational age, there was a lower need for intubation in the group that received a sustained inflation (29% vs 63%, *p* = 0.05), but this was not a pre-specified analysis [20]. In contrast to those promising results in moderately or extremely prematurely born infants, in infants born at 34–36 weeks of gestational age there were no significant differences in the need for respiratory support, the incidence of neonatal intensive care unit (NICU) admission for respiratory distress or the total length of NICU stay between infants who received a 15-s sustained inflation and those who received standard resuscitation according to the American Association of Pediatrics guidelines (drying, stimulation and, if inadequate respiratory effort or a heart rate < 100 bpm, positive pressure ventilation at 40–60 inflations per minute) [21].

There have been concerns raised regarding potential adverse effects of sustained inflations, in particular relating to air leak, intraventricular haemorrhage (IVH) and patent ductus arteriosus [22, 23]. Non-significant increases in IVH in infants born at < 28 weeks of gestational age [16] and non-significant increases in pneumothorax in infants born at < 34 weeks of gestational age have been reported in those exposed to sustained inflations [19]. A meta-analysis of four small trials [24], however, showed no significant differences in rates of pneumothorax or of IVH. A subsequent study of prematurely born infants showed a non-significant decrease in pneumothorax with a 15-s sustained inflation compared to ventilation with a self-inflating bag [25]. The peak inspiratory pressures, however, used in the bag-mask ventilation were up to 40 cmH_2_O, whereas those used in the sustained inflation group were 30 cmH_2_O [25]. The meta-analysis [24] also reported that in the sustained inflation group there was a higher proportion of infants who required treatment (medical or surgical) for a patent ductus arteriosus (relative risk = 1.27 [95% confidence interval = 1.05–1.54]). Cerebral blood flow, as assessed by near-infrared spectroscopy, however, was more stable in preterm infants resuscitated with a sustained inflation compared to in those who were resuscitated without a sustained inflation [26–28]. In addition, in prematurely born infants, a 5-s inflation compared to a 2-s inflation was not associated with higher levels of inflammatory cytokines (IL-6, IL-10, IL-1β, and TNF-α) in bronchoalveolar fluid [29].

Resuscitation guidelines from the USA, UK and Europe suggest that sustained inflations should be researched further [30, 31]. To date, there are no studies comparing sustained inflations to the current UK practice of the first five inflations lasting 2–3 s. In addition, there has only been one study in which a respiratory function monitor was used to assess the physiological effects of a sustained inflation. Sustained inflations were shown not to be effective in producing tidal volumes > 2.5 mL/kg or generating a functional residual capacity unless there was spontaneous respiratory effort during the inflation, but there was no comparison to other methods of resuscitation [32]. The aim of this study, therefore, is to determine whether a 15-s sustained inflation or five inflations each of 2–3 s duration is more effective at stimulating respiratory efforts in prematurely born infants. The primary outcome is the minute volume in the first minute of resuscitation and the secondary outcomes are the time to the first active inflation (inspiration occurring during an inflation) and end tidal carbon dioxide (ETCO_2_) levels in the first minute.

## Methods

This is a non-blinded, randomised superiority trial. Infants are allocated to parallel groups in a 1:1 ratio.

### Setting

A single tertiary neonatal unit at King’s College Hospital, London, UK

### Inclusion criteria

Inclusion criteria are as follows:

1. Infants delivered at < 34 weeks of gestational age.
2. The clinical team present before delivery and trained both in the use of the respiratory function monitor and to deliver sustained inflations.

### Exclusion criteria

1. Major congenital abnormalities
2. Refusal of consent for the data to be analysed (see later).

### Recruitment

This study is conducted as emergency research without prior consent. As soon as possible after delivery, parents are approached to inform them about the study and to ask them for written, informed consent for retention and analysis of the respiratory function data. The study and this retrospective consent approach were approved by the Health Research Authority and by the London-Riverside NHS Research Ethics Committee. Posters giving information about the study are displayed in the antenatal ward and on the delivery suite, and parents of eligible infants are encouraged to contact the researchers before delivery if they would like to discuss the study.

### Randomisation

The randomisation sequence is generated using a computerised random number generator and concealed in sealed, opaque envelopes. This is done by a person independent of the research team who is not involved in the study.

### Enrolment

Infants will be enrolled in the study by the clinical team attending the delivery. Infants will be deemed to be enrolled if the monitoring equipment is taken to the delivery, the infant is randomised into the study by opening the next envelope, and they do indeed require stabilisation.

### Blinding

The study is not blinded. Decisions regarding ongoing care, for example, the need for intubation and mechanical ventilation are made by the clinical team alone according to the unit’s guidelines.

### Intervention

**Fig. 1:**
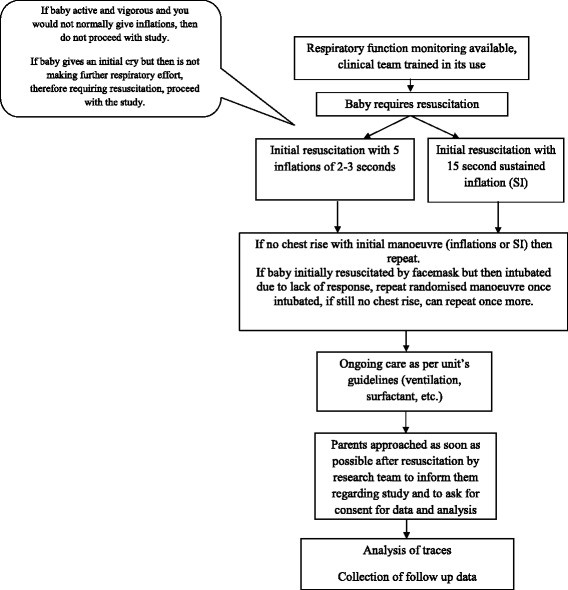
Trial *flowchart* (Protocol v3, 31/8/16)

At delivery, eligible infants are randomised either to receive five inflations each of 2–3 s duration as per the current UK Neonatal Life Support guidelines [2] or a 15-s sustained inflation (Fig. 1). Resuscitation is performed using a t-piece device (Neopuff Infant Resuscitator, Fisher & Paykel Healthcare, Auckland, New Zealand). Both the sustained inflation and the five 2–3 s inflations are performed using a peak inspiratory pressure of 25 cmH_2_O and PEEP of 5 cm H_2_O as per unit guidelines. As per unit guidelines, PEEP of 5 cmH_2_O is provided between inflations and between sets of inflations. Either the sustained inflations or the five inflation breaths may be repeated once and, if the baby is initially resuscitated by facemask and subsequently intubated, the sustained inflations or five 2–3 s inflations as per randomisation may be repeated twice if necessary. All trainees are trained to deliver the sustained inflation on a mannequin and using the respiratory function monitoring equipment.

The resuscitation is recorded using a respiratory function monitor (NM3 respiratory profile monitor; Philips Respironics, CT, USA). The monitor records flow, pressure, ETCO_2_ and tidal volume from a combined flow and carbon dioxide sensor that is placed between the Neopuff device and the endotracheal tube or facemask. Heart rate and oxygen saturations are recorded on the same device from a saturation probe applied to the right wrist (Masimo SET; Masimo Corporation, Irvine, CA, USA). Data are displayed and recorded on a personal computer running customised Spectra software (3.0.1.4) (Grove Medical, London, UK).

### Primary outcome measure

The primary outcome measure is the minute volume in the first minute of ventilation

### Secondary outcomes

The secondary outcome measures are the time to the first active inflation and the ETCO_2_ level in the first minute of resuscitation.

### Sample size

We have previously demonstrated that active compared to passive inflations during resuscitation at least double expiratory tidal volumes and ETCO_2_ levels [3]. During resuscitation with inflations of 1–3 s in duration, all infants had an active inflation by 50 s, but the active inflation occurred earlier in those who received longer rather than shorter inflations. The standard deviation of the minute volume was 71 mL/min/kg, which was 25% of the maximum minute volume achieved [3]. We, therefore, postulate that over the first minute of resuscitation the total expiratory tidal volume (minute volume) will be 25% higher in the sustained inflation group. Randomisation of 40 infants allows us to detect a difference of 71 mL/min/kg between the two groups with 90% power at the 5% significance level.

### Data collection

Demographic and outcome data will be collected from the clinical records and recorded under a unique study number on a password-protected computer. The gestational age, birthweight, sex, exposure to antenatal steroids, presence of chorioamnionitis, mode of delivery, need for mechanical ventilation in the first 48 h after birth, total duration of mechanical ventilation, duration of oxygen therapy, pneumothorax, BPD (oxygen dependency at 28 days after birth), patent ductus arteriosus and the findings on the first cranial ultrasound scan will be recorded for each infant. Traces from the respiratory function monitor will be analysed by the investigators to determine minute volume, ETCO_2_ level and the time to the first active inflation using customised Spectra software (3.0.1.4) (Grove Medical, London, UK).

**Fig. 2:**
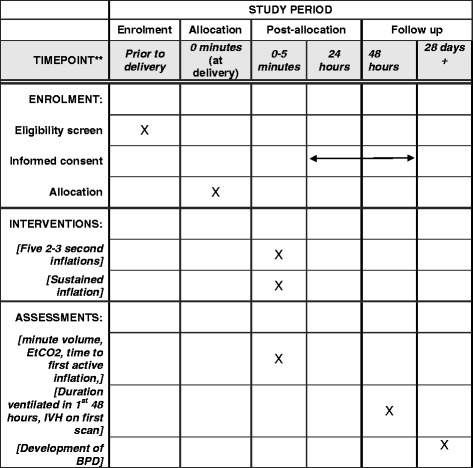
SPIRIT figure of trial interventions and timings

The order of study events is detailed in the Standard Protocol Items: Recommendations for Interventional Trials (SPIRIT) figure in Fig. 2. A SPIRIT checklist is also provided as an Additional file 1.

### Statistical analysis

Analysis will be conducted on an intention-to-treat basis. The data will be assessed for normality and if found not to have a normal distribution, non-parametric statistics will be used to assess if there are any significant differences in outcomes between the two groups. Analysis will be undertaken using IBM SPSS Statistics for Windows, version 22.

### Safety

Reports of related and unexpected serious adverse events will be submitted to the Research Ethics Committee within 15 days of the chief investigator becoming aware of the event. The parents will be informed of any events as soon as possible and be provided with an opportunity to meet with clinical and research team. Although meta-analysis of previous trials has not demonstrated an increased risk, the research team will specifically monitor for incidence of pneumothoraces as a potential identifiable adverse event. The results of any reports or investigations relating to the events will also be communicated to the parents in writing.

## Discussion

This study will compare the effectiveness of a sustained inflation to five 2–3 s inflations which are currently recommended by the Resuscitation Council (UK) Neonatal Life Support Guidelines [2]. Physiological outcomes have been chosen as they will allow demonstration of significant differences between the two interventions with a relatively small sample size and hence in a short time span.

This study is performed as emergency research without prior consent, but with approval from an ethics committee. Preterm delivery may occur with little or no advanced warning, so it can be difficult to obtain fully informed consent before delivery. Furthermore, if consent is sought antenatally, many mothers will consent for studies for which their infants are never eligible [33]. An alternative approach is to waive prospective informed consent and to enrol eligible infants as soon as possible. A study [34] surveyed international resuscitation scientists at an international resuscitation research workshop regarding their views on retrospective or deferred consent. There was a 78% response rate and respondents came from 15 countries. Ninety-one percent of respondents agreed to the statement ‘Enrolling subjects for delivery room resuscitation without antenatal informed consent is an acceptable trade off between respect for persons for enrolled subjects and potential for all sick new borns’. The investigators were concerned about the scientific limitations of the previous studies using antenatal consent and thought the most common methodological flaw in such studies was selection bias.

In the event that this study concludes that the traditional five 2–3 s inflations are superior to the sustained inflations, the study would have significant impact by solidifying the scientific background of the intervention that is currently used in the UK. Given the potential link of the mode of resuscitation and later development of chronic respiratory morbidity [35], this study could possibly unravel an association between the mode of resuscitation and later, longer-term respiratory outcomes such as the duration of mechanical ventilation and the diagnosis of BPD.

### Trial status

At the time of submission, this trial has been approved by the NHS Research Ethics Committee and the Health Research Authority and is recruiting participants.

## Additional file

Additional file 1: SPIRIT 2013 Checklist: Recommended items to address in a clinical trial protocol and related documents. (DOC 122 kb)

## Abbreviations

BPD: Bronchopulmonary dysplasia
CO_2_: Carbon dioxide
CPAP: Continuous positive airways pressure
ETCO_2_: End tidal carbon dioxide
FiO_2_: Fraction of inspired oxygen concentration
FRC: Functional residual capacity
IVH: Intraventricular haemorrhage
NICU: Neonatal intensive care centre
PEEP: Positive end expiratory pressure
